# Influence of vitamin D on key bacterial taxa in infant microbiota in the KOALA Birth Cohort Study

**DOI:** 10.1371/journal.pone.0188011

**Published:** 2017-11-09

**Authors:** Chris E. Talsness, John Penders, Eugène H. J. M. Jansen, Jan Damoiseaux, Carel Thijs, Monique Mommers

**Affiliations:** 1 Department of Epidemiology, Care and Public Health Research Institute (CAPHRI), Maastricht University, Maastricht, Netherlands; 2 Department of Medical Microbiology, Care and Public Health Research Institute (CAPHRI), Maastricht University Medical Center, Maastricht, Netherlands; 3 Centre for Health Protection, National Institute for Public Health and the Environment, Bilthoven, Netherlands; 4 Laboratory for Clinical Immunology, Maastricht University Medical Center, Maastricht, Netherlands; Mayo Clinic Rochester, UNITED STATES

## Abstract

Vitamin D has immunomodulatory properties giving it the potential to affect microbial colonization of the intestinal tract. We investigated whether maternal vitamin D supplemention, maternal plasma 25-hydroxyvitamin D concentration, or direct supplementation of the infant influences key bacterial taxa within microbiota of one month old infants. Infant and maternal vitamin D supplement use was ascertained via questionnaires. Maternal plasma 25-hydroxyvitamin D was determined at approximately the 36^th^ week of pregnancy. In 913 one month old infants in the prospective KOALA Birth Cohort Study, fecal *Bifidobacterium* spp., *Escherichia coli*, *Clostridium difficile*, *Bacteroides fragilis* group, *Lactobacillus* spp. and total bacteria were quantified with real-time polymerase chain reaction assays targeting 16S rRNA gene sequences. The association between vitamin D exposure and prevalence or abundance of a specific bacterial group or species was analyzed using logistic or linear regression, respectively. There was a statistically significant negative linear trend between counts of *Bifidobacterium* spp. and levels of maternal vitamin D supplementation and maternal 25-hydroxyvitamin D quintiles, respectively. In addition, a positive linear trend between quintile groups and *B*. *fragilis* group counts was observed. Lower counts of *C*. *difficile* were associated with vitamin D supplementation of breast fed infants whose mothers were more likely to adhere to an alternative lifestyle in terms of, *e*.*g*., dietary habits. These data suggest that vitamin D influences the abundance of several key bacterial taxa within the infant microbiota. Given that intestinal microbiotic homeostasis may be an important factor in the prevention of immune mediated diseases and that vitamin D status is a modifiable factor, further investigation of the impact of postnatal vitamin D supplementation should be conducted in older infants.

## Introduction

The intestinal microbiota plays a decisive role in directing immune development early post-partum and shaping immune responses throughout life [1]. Studies demonstrate that changes in microbiota are associated with a variety of immune mediated diseases [2]. The importance of intestinal bacterial homeostasis in the etiology of diseases emphasizes the need to identify modifiable factors determining intestinal bacterial composition.

As the intestinal microbiota influences immune function, so can the mucosal immune system regulate the composition of the microbiota [3]. The host can control bacterial composition via the production of antimicrobial peptides (defensins and cathelicidin) along the epithelial surface [4]. Vitamin D is a known regulator of antimicrobial peptide expression [5] and has been shown to up-regulate antimicrobial peptide gene expression in a variety of cell types [6–8], including colonic cells [9]. In addition, a study of sepsis patients demonstrated a positive association between vitamin D status and plasma cathelicidin concentrations [10]. Vitamin D also promotes non-inflammatory states by directing immune responses in favor of tolerance by inhibiting dendritic cell maturation and differentiation [11]. Minimizing intestinal inflammation promotes homeostasis of the microbiota as inflammation is a mechanism whereby pathogenic bacteria can overcome colonization resistance by resident bacteria [12].

Vitamin D status, therefore, has the potential to influence the intestinal microbiota due to its promotion of anti-inflammatory responses by the immune system and inhibition of infections. Given that vitamin D status is a modifiable factor which may influence intestinal microbiotic homeostasis, which in turn is indicated to be important in the prevention of immune mediated diseases, we investigated whether maternal use of multivitamin supplements containing vitamin D, maternal vitamin D status (25 hydroxyvitamin D (25(OH)D), or administration of vitamin D supplement to the baby influences the presence and abundance of several key bacterial taxa of the infant gut microbiota.

## Methods

### Subjects

The participants were recruited from the prospective KOALA Birth Cohort Study previously described in detail elsewhere [13]. Briefly, pregnant women were enrolled between the 14^th^ and 18^th^ week of pregnancy from October 2000 until December 2002. Mothers (n = 2343) were recruited from an ongoing prospective Dutch cohort study on pregnancy-related pelvic girdle pain (conventional recruitment group) and through Steiner schools, organic food shops, and anthroposophic doctors, midwives and magazines (alternative recruitment group)(n = 491). Most of the women in this latter group were considered to have an alternative lifestyle, in terms of dietary habits, child rearing practices,vaccination schemes and/or use of antibiotics. In the alternative recruitment group 73% of the women were vegetarians, or had organic or macrobiotic dietary habits, compared to 7% in the conventional recruitment group. Comparing the alternative recruitment group to the conventional recruitment group, the alternative recruitment group had a greater proportion of women with a high educational level (73% vs. 43%) and who exclusively breast fed in the first 6 months of the child’s life (56% vs. 20%). Moreover, fewer women in the alternative recruitment group smoked during pregnancy (1% vs. 8%) and adhered to conventional vaccination schemes (46% vs. 84%), and their infants were less likely to be exposed to environmental tobacco smoke (2% vs. 14%) [13].

Written informed consent was obtained from the parents and ethical approval was granted by the Medical Ethics Committee of the University Hospital of Maastricht.

Beginning in January 2002, consent was also requested to obtain a maternal blood sample at approximately the 36^th^ week of gestation and an infant fecal sample at one month of age for microbial analyses. Data regarding possible confounders and vitamin D exposure through supplement use was assessed from questionnaires sent to all parents at 34 weeks of gestation, the time of fecal sampling and 3 months postpartum, respectively.

Inclusion criteria for the current study included availability of an infant fecal sample and the accompanying questionnaire data at one month of age, and written informed consent. Mother—child pairs were excluded for reasons of prematurity (<37 weeks), Down’s Syndrome, antibiotic, antimycotic or probiotic use by the infant or antibiotic use by a breast feeding mother prior to fecal sampling. Further exclusions were made for unsuitable infant fecal samples according to the following criteria: samples weighing less than 1g, or samples collected before 3 weeks or after 6 weeks of age.

### Maternal vitamin D supplement use

Maternal vitamin D intake through use of multivitamin supplements containing vitamin D was calculated as previously described [14]. Vitamin D dosage in the supplements was estimated according to an overview of vitamin preparations [15] and intake was categorized as none (0 μg), <10 μg/day or ≥ 10 μg/day. Diet and sunlight exposure were not considered as 25(OH)D concentrations were measured in mothers providing an estimation of exposure based on all sources and only “yellow fat spreads” were fortified with vitamin D in the Netherlands at the time of the study.

### Infant vitamin D supplement use

Vitamin D supplementation of the infant was categorized as yes or no. Thirty-seven children received a vitamin A-D supplement and were included in the group of vitamin D supplemented children. Sunlight exposure was not considered because one month old babies are typically covered and not placed in direct sunlight.

### Maternal plasma 25(OH)D concentration

Blood samples were immediately centrifuged, transported at 4°C and stored at -80°C in a biobank until further analysis. Plasma concentrations of 25(OH)D were measured with an ELISA kit from Immuno diagnostic Systems (Boldon, UK) according to the manufacturer’s instructions.

### Fecal sample collection, DNA isolation and microbial analysis

The collection of fecal samples has been described in detail elsewhere [16]. Briefly, parents placed a sanitary napkin in the diaper to prevent absorption of the feces and sent the sample (without the addition of preservation media) on the same day to the laboratory by post. Samples were diluted tenfold in peptone-water (Oxoid CM0009) containing 20% v/v glycerol (Merck, Darmstadt, Germany) and stored at -20°C until analyzed. DNA was isolated from the feces using a method previously described in detail [16] based upon bead-beating followed by the use of the QIAamp DNA stool mini kit (Qiagen, Hilden, Germany) according to the manufacturer’s instructions. Quantification of *Bifidobacterium* spp, *Escherichia coli*, *Clostridium difficile*, *Bacteroides fragilis* group, *Lactobacillus* spp and total bacteria was achieved with real-time polymerase chain reaction assays targeting 16S ribosomal ribonucleic acid (rRNA) gene sequences. The 5’-nuclease technique was used to detect *Bifidobacterium* spp, *E*. *coli*, *C*. *difficile*. and the *B*. *fragilis* group. *Lactobacillus* spp and total bacteria were detected with SYBR Green 1 (Bio-Rad Laboratories, Hercules, CA). Specifics regarding the primers, probes and PCR conditions have been presented elsewhere [16]. Bacterial counts were determined from the threshold cycle values by using the constructed standard curves. The outcomes investigated were prevalence of colonization by a specific bacterial species or group which was expressed as yes or no, and counts of bacteria defined as log_10_ colony forming units (CFU)/g feces of colonized infants.

### Statistical analyses

The association between vitamin D exposure and prevalence or exposure and abundance of specific groups or species (log_10_ CFU/g wet weight faeces) only in infants who were colonized with the bacteria under study was analyzed using logistic or linear regression, respectively.

The potential confounders included in the models can be found in the tables depicting the results of the analyses. In the analysis regarding supplementation of the infant, both bottle fed and combination fed (bottle and breast milk) children were excluded because commercial formulas contain vitamin D and the amount of vitamin D supplementation would vary depending on the volume of formula consumed. The analyses regarding maternal vitamin D supplementation and maternal 25(OH)D quintiles were performed on the entire population because the exposure of interest was prenatal, *i*.*e*. occurring before the introduction of either formula or breast milk. Cases with missing data were excluded when the exposure, outcome or confounder variable was missing. Due to the study design, we tested for possible interaction between vitamin D exposure and recruitment group (conventional/alternative). When the p value for the interaction term was < 0.05, stratification by recruitment group was undertaken to account for effect modification. The categorical variables, quintiles of maternal 25(OH)D and amount of maternal vitamin D supplementation, were transformed into interval variables to conduct the tests for interaction, as well as the tests for linear trend performed with linear regression. An unpaired t test was conducted to compare the mean maternal 25(OH)D concentrations between mothers who used supplements and those who did not. Data analysis was performed using SPSS 20.0 for Windows (Chicago, IL) and p<0.05 was considered statistically significant. False disovery rate (FDR) was applied to the ten tests for linear trend to correct for multiple testing (q = 0.05).

## Results

### Study population

There were 1176 potentially eligible participants from the KOALA cohort (children with a fecal sample). For technical reasons, 144 infants were excluded because the fecal sample: weighed < 1 g (n = 65), was collected before 3 weeks or after 6 weeks of age (n = 25), or the questionnaire accompanying the fecal sample was not submitted (n = 25). Other reasons for exclusion from the analysis included premature birth (< 37 weeks) (n = 14), Down’s Syndrome (n = 3), antibiotic or antimycotic use by the infant or antibiotic use by a breast feeding mother prior to fecal sampling (n = 95), lack of information about antimicrobial use (n = 4), uncertainity concerning antimicrobial use (n = 1) and administration of probiotics to the child during the first month of life (n = 2), resulting in 913 participants in the study. The analyses concerning vitamin D supplementation of the breast fed infant included 616 individuals after omitting infants receiving formula (n = 208), both breast milk and formula (n = 87) and lack of information regarding feeding (n = 2).

The prevalence of colonization of neonatal fecal samples with the studied bacteria was 98.7% (901/913) for bifidobacteria, 88.7% (807/910) for *E*. *coli*, 81.5% (744/913) for *B*. *fragilis* group, 25.1% (229/913) for *C*. *difficile* and 32% (292/913) for lactobacilli.

Table 1 depicts the numbers of participants according to: vitamin D supplement administration to the infant, maternal use of multivitamin supplements containing vitamin D, and maternal 25(OH)D quintiles and other characteristics. The characteristics of the breast fed children were fairly similar between the children receiving vitamin D supplementation and those who did not, except that mothers who were recruited in the conventional recruitment group were more likely to give their child vitamin D supplementation than those in the alternative recruitment group. There was also a minor difference in that parents with more children tended not to supplement their infant with vitamin D. In terms of use of multivitamin supplements containing vitamin D, by the mother, those in the conventional group were more represented in the none and highest categories of supplementation with approximately equal representation of recruitment groups occuring in the < 10 μg category. Finally, there was an expected difference among the quintiles in terms of season of blood sampling in that the higher quintiles included a lower percentage of mothers giving blood during the winter season and a higher percentage in the summer.

**Table 1:** Characteristics of the study population (n = 913).

	Maternal multivitamin supplementation containing vitamin D (n = 913)			Maternal plasma 25(OH)D nmol/L (n = 892)					Infant Vitamin D Supplementation^§^(n = 616)	
	none	< 10 μg	≥ 10 μg	Q 1	Q 2	Q 3	Q 4	Q 5	Yes	No
				7.7–27.3^#^	27.4–37.3^#^	37.4–47.8^#^	47.9–59.9^#^	60.0–126.4^#^		
	n = 350	n = 144	n = 419	21.4 (4.3)^&^	32.3 (2.9)^&^	43.0 (3.2)^&^	53.3 (3.5)^&^	71.9 (10.9)^&^	n = 441	n = 165
	n (%)	n (%)	n (%)	n (%)	n (%)	n (%)	n (%)	n (%)	n (%)	n (%)
**Mode and Place of Delivery**										
Vaginal / Home	171 (48.9)	71 (49.3)	188 (44.9)	80 (44.9)	87 (48.6)	80 (44.7)	92 (51.7)	78 (43.8)	245 (55.6)	93 (56.3)
Vaginal / Hospital	137 (39.1)	56 (38.9)	174 (41.5)	68 (38.2)	74 (41.3)	77 (43.0)	63 (35.4)	78 (43.8)	157 (35.6)	58 (35.2)
C-section / Hospital	36 (10.3)	12 (8.3)	47 (11.2)	21 (11.8)	15 (8.4)	19 (10.6)	21 (11.8)	18 (10.1)	39 (8.8)	14 (8.5)
Missing	6 (1.7)	5 (3.5)	10 (2.4)	9 (5.1)	3 (1.7)	3 (1.7)	2 (1.1)	4 (2.2)	0	0
**Number of Siblings**										
None	130 (37.1)	68 (47.2)	166 (39.6)	71 (39.9)	71 (39.7)	73 (40.8)	83 (46.7)	61 (34.3)	185 (42)	46 (27.9)
1	153 (43.7)	51 (35.4)	197 (47.0)	79 (44.4)	79 (44.1)	82 (45.8)	72 (40.4)	77 (43.2)	189 (42.8)	76 (46.1)
2 or more	66 (18.9)	25 (17.4)	56 (13.4)	28 (15.7)	28 (15.6)	24 (13.4)	23 (12.9)	40 (22.5)	66 (15)	43 (26)
Missing	1 (0.3)	0	0	0	1 (0.6)	0	0	0	1 (0.2)	0
**Recruitment Group**										
Conventional	233 (66.6)	66 (45.8)	322 (76.8)	114 (64)	117 (65.4)	121 (67.6)	129 (72.5)	132 (74.2)	298 (67.6)	54 (32.7)
Alternative	117 (33.4)	78 (54.2)	97 (23.2)	64 (36)	62 (34.6)	58 (32.4)	49 (27.5)	46 (25.8)	143 (32.4)	111 (67.3)
**Maternal Vitamin D Quintile**										
1	104 (29.7)	30 (20.8)	44 (10.5)						64 (14.5)	40 (24.2)
2	83 (23.7)	30 (20.8)	66 (15.8)						87 (19.7)	38 (23)
3	59 (16.9)	25 (17.4)	95 (22.7)						88 (20)	24 (14.5)
4	60 (17.1)	22 (15.3)	96 (22.9)						98 (22.2)	28 (17)
5	38 (10.9)	30 (20.8)	110 (26.2)						96 (21.8)	29 (17.7)
Missing	6 (1.7)	7 (4.9)	8 (1.9)						8 (1.8)	6 (3.6)
**Sex**										
Male	196 (56)	63 (43.8)	200 (47.7)	102 (57.3)	87 (48.6)	93 (52)	78 (43.8)	89 (50)	219 (49.7)	81 (49.1)
Female	154 (44)	81 (56.2)	219 (52.3)	76 (42.7)	92 (51.4)	86 (48)	100 (56.2)	89 (50)	222 (50.3)	84 (50.9)
**Vaginitis (Last Month of Pregnancy)**										
No	319 (91.2)	123 (85.4)	372 (88.8)	156 (87.6)	161 (89.9)	163 (91)	162 (91)	153 (86)	409 (92.7)	143 (86.7)
Yes	25 (7.1)	16 (11.1)	37 (8.8)	13 (7.3)	15 (8.4)	13 (7.3)	14 (7.9)	21 (11.8)	32 (7.3)	22 (13.3)
Missing	6 (1.7)	5 (3.5)	10 (2.4)	9 (5.1)	3 (1.7)	3 (1.7)	2 (1.1)	4 (2.2)	0	0
**Mode of Infant Feeding**										
Breast Feeding	243 (69.4)	105 (72.9)	268 (64.0)	107 (60.1)	125 (69.8)	114 (63.7)	128 (71.9)	128 (71.9)		
Bottle Feeding	80 (22.9)	25 (17.4)	103 (24.6)	52 (29.2)	39 (21.8)	50 (27.9)	29 (16.3)	34 (19.1)		
Combination Feeding	26 (7.4)	14 (9.7)	47 (11.2)	19 (10.7)	15 (8.4)	15 (8.4)	21 (11.8)	15 (8.4)		
Missing	1 (0.3)	0	1 (0.2)	0	0	0	0	1 (0.6)		
**Season of Maternal Blood Sampling**										
Winter				89 (50)	69 (38.6)	49 (27.4)	41 (23)	22 (12.4)		
Spring				54 (30.3)	51 (28.5)	46 (25.7)	31 (17.4)	16 (8.9)		
Summer				8 (4.5)	23 (12.8)	40 (22.3)	66 (37.1)	82 (46.1)		
Autumn				27 (15.2)	36 (20.1)	44 (24.6)	40 (22.5)	58 (32.6)		

^§^ only breast fed children;

^#^ range;

^&^ mean (SD)

### Maternal 25(OH)D and use of multivitamin supplements containing vitamin D

Maternal 25(OH)D concentrations of the entire group ranged from 7.7 to 126.3 nmol/L with a mean of 44.3, SD 18.3 nmol/L. The relationship between maternal 25(OH)D and maternal use of multivitamin supplements containing vitamin D is shown in Table 2. During seasons of both high and low cutaneous production of vitamin D, the mean maternal plasma 25(OH)D concentrations were significantly greater in mothers who used vitamin D containing supplements compared to those who did not. Use of multivitamin supplements containing vitamin D and maternal obesity were associated with maternal 25(OH)D level during both low and high seasons, while recruitment group was significantly associated with 25(OH)D concentration only during the high season.

**Table 2:** Mean maternal 25(OH)D concentration according to season of blood sampling and use of multivitamin supplements containing vitamin D.

	Blood sampling in high season of cutaneous vitamin D production	Blood sampling in low season of cutaneous vitamin D production
**Maternal use of multivitamin supplement containing vitamin D**	**Maternal Plasma 25(OH)D nmol/L**	**Maternal Plasma 25(OH)D nmol/L**
No (mean (SD))	45.2 (17.0)	30.1 (13.5)
Yes (mean (SD))	53.3 (17.8)	40.9 (16.1)
p value^&^	**p = 0.000**	**p = 0.000**
**Model** #	**Beta (95% CI)**	**Beta (95% CI)**
Constant	52.7 (47.7, 57.7)	34.6 (29.5, 39.7)
Maternal use of multivitamin supplement containing vitamin D (yes *vs*. no)	**7.8 (4.7, 10.9)**	**10.7 (7.6, 13.8)**
Pre-pregnancy overweight *vs*. not overweight	**-9.8 (-14.5, -5.0)**	**-5.4 (-10.8, -0.05)**
Alternative *vs*. Conventional recruitment group	**-4.6 (-7.8, -1.4)**	-3.1 (-6.4, 0.3)

High season = April–October, Low Season = November–March; Overweight = Body mass index > 90^th^ percentile;

^&^ unpaired t-test;

^#^ regression coefficients (Beta) from multivariable linear regression analysis indicate the difference in maternal plasma 25(OH)D in nmol/L between the group of mothers taking multivitamin supplement containing vitamin D and those who did not while controlling for the other factors in the model

### Maternal vitamin D intake through use of multivitamin supplements containing vitamin D

Maternal use of multivitamin supplements containing vitamin D was not associated with the prevalence of any of the bacteria in both the unadjusted and adjusted analyses (data not shown). There was a statistically significant negative linear trend between categories of maternal vitamin D supplementation and counts of *Bifidobacterium* spp. and *C*. *difficile*, respectively (Table 3)(for a visualisation of the data in univariable boxplots see also S1 Fig). The trend for bifidobacteria remained statistically significant following correction for multiple testing using the FDR.

**Table 3:** Association between maternal use of multivitamin supplements containing vitamin D and log_10_ colony forming units of bacterial species or groups in infants colonized with the respective bacteria.

Maternal use of multivitamin supplements containing vitamin D				
Maternal multivitamin supplementation containing vitamin D	Bacteria	^&^n	Unadjusted B (95% CI)	^#^Adjusted B (95% CI)
none	***Bifidobacterium* spp.**	346	0 (reference)	0 (reference)
< 10 μg		143	**-0.20 (-0.38, -0.02)**	-0.13 (-0.31, 0.04)
≥ 10 μg		412	**-0.15 (-0.28, -0.02)**	**-0.16 (-0.29, -0.04)**
P for linear trend				p = 0.012*
none	***Escherichia coli***	306	0 (reference)	0 (reference)
< 10 μg		127	-0.11 (-0.38, 0.15)	-0.02 (-0.26, 0.23)
≥ 10 μg		374	-0.10 (-0.30, 0.09)	-0.09 (-0.27, 0.09)
P for linear trend				p = 0.329
none	***Bacteroides fragilis* group**	281	0 (reference)	0 (reference)
< 10 μg		117	0.11 (-0.18, 0.40)	0.18 (-0.06, 0.41)
≥ 10 μg		346	-0.02 (-0.23, 0.20)	0.01 (-0.17, 0.18)
P for linear trend				p = 0.997
none	***Clostridium difficile***	84	0 (reference)	0 (reference)
< 10 μg		35	-0.46 (-1.25, 0.33)	-0.68 (-1.45, 0.08)
≥ 10 μg		110	-0.46 (-1.03, 0.10)	**-0.59 (-1.14, -0.04)**
P for linear trend				**p = 0.038**
none	***Lactobacillus* spp.**	116	0 (reference)	0 (reference)
< 10 μg		48	0.02 (-0.21, 0.24)	0.01 (-0.20, 0.22)
≥ 10 μg		128	-0.01 (-0.17, 0.16)	0.03 (-0.12, 0.19)
P for linear trend				p = 0.684

^&^ number of infants colonized;

^#^ adjusted for: place and mode of delivery, number of siblings, recruitment group, total bacterial counts, sex, vaginitis during the last month of pregnancy and mode of infant nutrition;

* remained statistically significant after correction with FDR (q < 0.05)

### Maternal plasma 25-hydroxyvitamin D quintiles

The likelihood of colonization with *B*. *fragilis* was increased in the second 25(OH)D quintile compared to the first (OR = 2.30; 95% CI: 1.29, 4.13 and adjOR = 2.17; 95% CI 1.16, 4.05). A negative linear trend between quintile groups and bifidobacteria counts and a positive linear trend between quintile groups and *B*. *fragilis* group counts were observed (Table 4) (for a visualisation of the data in univariable boxplots see also S2 Fig). Both trends remained statistically significant following correction for multiple testing using the FDR.

**Table 4:** Association between maternal 25(OH)D levels during pregnancy and log_10_ colony forming units of bacterial species or groups in infants colonized with the respective bacteria.

Maternal 25(OH)D	Bacteria	^&^n	Unadjusted B (95% CI)	^#^Adjusted B (95% CI)
Quintile 1	***Bifidobacterium* spp.**	176	0 (reference)	0 (reference)
Quintile 2		178	0.06 (-0.12, 0.25)	-0.02 (-0.20, 0.16)
Quintile 3		175	0.07 (-0.11, 0.26)	-0.01 (-0.20, 0.17)
Quintile 4		175	-0.09 (-0.27, 0.10)	-0.14 (-0.33, 0.06)
Quintile 5		176	**-0.19 (-0.38, -0.01)**	**-0.25 (-0.46, -0.05)**
p for linear trend				p = 0.010*
Quintile 1	***Escherichia coli***	155	0 (reference)	0 (reference)
Quintile 2		160	-0.06 (-0.34, 0.22)	-0.09 (-0.35, 0.17)
Quintile 3		160	0.04 (-0.24, 0.32)	-0.04 (-0.31, 0.22)
Quintile 4		156	-0.06 (-0.34, 0.22)	-0.06 (-0.33, 0.22)
Quintile 5		159	-0.04 (-0.32, 0.24)	-0.09 (-0.38, 0.19)
p for linear trend				p = 0.655
Quintile 1	***Bacteroides fragilis* group**	138	0 (reference)	0 (reference)
Quintile 2		159	0.15 (-0.15, 0.45)	0.06 (-0.19, 0.30)
Quintile 3		150	**0.42 (0.12, 0.72)**	**0.30 (0.05, 0.55)**
Quintile 4		139	0.29 (-0.02, 0.60)	0.24 (-0.02, 0.50)
Quintile 5		142	0.09 (-0.22, 0.39)	**0.28 (0.01, 0.55)**
P for linear trend				p = 0.014*
Quintile 1	***Clostridium difficile***	46	0 (reference)	0 (reference)
Quintile 2		42	-0.40 (-1.22, 0.43)	-0.34 (-1.16, 0.48)
Quintile 3		55	-0.43 (-1.20, 0.34)	-0.40 (-1.19, 0.40)
Quintile 4		42	-0.04 (-0.86, 0.79)	0.19 (-0.66, 1.04)
Quintile 5		41	-0.61 (-1.45, 0.22)	-0.30 (-1.18. 0.58)
P for linear trend				p = 0.949
Quintile 1	***Lactobacillus* spp.**	60	0 (reference)	0 (reference)
Quintile 2		68	0.01 (-0.21, 0.24)	0.01 (-0.21, 0.22)
Quintile 3		49	-0.11 (-0.35, 0.14)	-0.18 (-0.41, 0.06)
Quintile 4		51	0.02 (-0.22, 0.26)	0.04 (-0.20, 0.28)
Quintile 5		58	-0.06 (-0.30, 0.17)	0.08 (-0.17, 0.33)
P for linear trend				p = 0.459

^&^ number of children colonized;

^#^ Adjusted for: place and mode of delivery, number of siblings, recruitment group, total bacterial counts, sex, vaginitis during the last month of pregnancy, mode of infant nutrition and season of blood sampling; Quintile 5 is the highest quintile;

* remained statistically significant after correction with FDR (q < 0.05)

### Vitamin D supplementation of the infant

There was no influence of vitamin D supplementation of the infant during the first month of life on the prevalence of any of the bacterial species or groups (data not shown). Table 5 shows the association between vitamin D supplementation of the infant in the first month of life and the log_10_ CFUs of the different bacteria in children colonized with the respective bacterial group or species (for a visualisation of the data in univariable boxplots see also S3 Fig).

**Table 5:** Association between vitamin D supplementation of the infant and log_10_ colony forming units/g feces of bacterial species or groups in breast fed infants (n = 616) colonized with the respective bacteria.

Vitamin D Supplement Use of Breast Feeding Infants				
Vitamin D supplementation	Bacteria	^&^n	Unadjusted B (95% CI)	^#^Adjusted B (95% CI)
No use	***Bifidobacterium* spp.**	164	0 (reference)	0 (reference)
Use		435	-0.10 (-0.26, 0.05)	-0.12 (-0.28, 0.04)
No use	***Escherichia coli***	134	0 (reference)	0 (reference)
Use		385	0.02 (-0.24, 0.27)	-0.02 (-0.27, 0.24)
No use	***Bacteroides fragilis* group**	130	0 (reference)	0 (reference)
Use		348	0.05 (-0.21, 0.32)	-0.02 (-0.26, 0.21)
No use	***Clostridium difficile***	35	0 (reference)	0 (reference)
Use		95	-0.48 (-1.19, 0.23)	-0.40 (-1.15, 0.34)
No use	***Lactobacillus* spp.**	45	0 (reference)	0 (reference)
Use		132	-0.10 (-0.31, 0.11)	-0.15 (-0.37, 0.07)

^&^ number of children colonized;

^#^ adjusted for: place and mode of delivery, number of siblings, recruitment group, total bacterial counts, sex, vaginitis during the last month of pregnancy, and quintile of maternal 25(OH)D level.

Effect modification by recruitment group (alternative *vs* conventional) was observed for counts of *B*. *fragilis* group (p-value for interaction p = 0.036) and *C*. *difficile* (p-value for interaction p = 0.032). Following stratification by recruitment group, no effect was observed for *B*. *fragilis* in the conventional (adjB = 0.34; 95% CI: -0.02, 0.71) or alternative (B = -0.18; 95% CI: -0.50, 0.15) groups. Lower counts of *C*. *difficile* were associated with vitamin D supplementation in the alternative group (B = -1.45 log_10_ CFU/g feces; 95% CI: -2.63, -0.28) with no association found in the conventional group (B = 0.49; 95% CI: -0.71, 1.69).

## Discussion

The objective of this study was to investigate the potential of vitamin D to influence the presence and or abundance of several key bacterial taxa within the infant intestinal microbiota. We theorized that vitamin D could impact intestinal colonization due to its immunomodulatory effects [17,18]; namely, through its inhibition of intestinal infections via stimulated antimicrobial peptide production and promotion of anti-inflammatory responses by the immune system. The abundance of several bacterial taxa in infant fecal samples at one month after birth was shown to be associated with pre- or postnatal vitamin D exposure, supporting the postulation that vitamin D can influence the composition of the intestinal microbiota.

The intestinal mucosa is faced with the constant task of differentiating between commensal and pathogenic microbiota. Acceptance of commensal organisms is characterized by tolerance which is achieved by regulating innate and adaptive immune responses to dampen inflammation, whereas the latter is directed toward elimination of pathogens with accompanying tissue damage [19].

Vitamin D modulates innate immunity by regulating one of its main actors, antimicrobial peptides (AMP), which have a variety of functions not only including microbiocidal activity and chemotaxis of inflammatory immune cells [4]. It has been demonstrated that the level of expression of AMP can influence the relative composition of the intestinal microbiota in a transgenic mouse model for human α-defensin 5 (HD5) [20] and in mice deficient for murine α-defensins [21]. The function of vitamin D in regulating AMP production, therefore, suggests that vitamin D status could influence the composition of the intestinal microbiota.

Active vitamin D influences tolerance by inhibiting dendritic cell maturation and differentiation. This leads to increased secretion of interleukin 10 (IL-10), favoring the induction of regulatory T cells and their production of IL-10, over inflammatory T cell formation [11]. This type of regulated immune response mirrors the tactic employed by some commensal bacterial species in order to avoid elimination [12,22]. Minimizing intestinal inflammation promotes homeostasis of the microbiota as inflammation can serve as a mechanism whereby pathogenic bacteria can overcome colonization resistance by resident bacteria [12]. This is supported by a study [23] demonstrating that an avirulent strain of Salmonella, incapable of inciting inflammation, could only colonize the murine intestine in induced- colitis models or IL-10 knock-out mice. A state of vitamin D deficiency resulting in a pro-inflammatory state and increased susceptibility to intestinal infections, therefore, may lead to altered microbial colonization known as dysbiosis or disturbance of the balance between beneficial commensal and pathogenic species [24].

In summary, the role of vitamin D is most likely to regulate the immune response to maintain immune homeostasis [25] by balancing activation of tolerance and the innate immune system. Although vitamin D promotes immune tolerance, adequate adaptive immune response, as measured by IgG production in response to infection or vaccination, has been observed in patients with relatively high serum 25(OH)D levels, suggesting that vitamin D maintains immune stability by preventing excessive immune responses in either direction [26].

Investigations studying the effect of vitamin D status on the composition of the intestinal microbiota are few; one study was performed in vitamin D deficient mice and two investigations in humans examined the role of vitamin D as secondary questions. Compatible with our present study, the results of these three investigations suggest that vitamin D may play a role in shaping the intestinal microbiota. In a mouse model of induced colitis, vitamin D deficient mice were shown to have decreased colonic antimicrobial activity (angiogenin-4 protein) and higher levels of 16S rDNA in colonic tissue (bacterial infiltration) compared to vitamin D sufficient mice [27]. In humans, a study investigating variation in diet and microbial composition of the intestinal tract between 52 African Americans and 46 Caucasian Americans found discordant vitamin D supplementation practices and differences between the counts of *Bacteroidetes* in the fecal samples between the two groups [28]. In a cross sectional analysis of 98 adults examining enterotype partitioning in relation to nutrient consumption (food frequency questionnaire), vitamin D supplement use and vitamin D from dairy sources were found to be associated with enterotypes. This finding, however, was no longer statistically significant following correction for multiple testing using the FDR [29].

Here we report on an association between prenatal vitamin D exposure and the abundance of several key bacterial taxa in the infant intestinal microbiota. The influence of direct vitamin D supplementation of the child differed between the two recruitment groups suggesting that lifestyle factors are modulating the association between vitamin D and the intestinal bacterial taxa. These two recruitment groups differ with respect to dietary habits, e.g., those in the alternative group tend to follow an organic or biodynamically produced diet with a higher intake of fermented legume and cereal products like tofu and seitan, and restricted use of antibiotics and vaccinations [13]. The latter differences, however, do not pertain to this study as antibiotic use was a ground for exclusion and vaccinations have not yet begun in one month olds. Maternal diet or other unidentified factors, *e*.*g*., health issues leading to the choice of an alternative lifestyle, could be modifying the effect of vitamin D on the intestinal bacteria and, therefore, recruitment group was adjusted for in the multivariable analyses.

A strength of our study is that we investigated the influence of both maternal supplementation and that of the child. For the latter, we were able to adjust for the maternal 25(OH)D concentration during the 36^th^ week of pregnancy. This is important because the 25(OH)D status of the one month old infant is still highly influenced by the maternal 25(OH)D concentration due to the approximately three week half life of circulating 25(OH)D [30]. In a future study, it would be preferable to investigate the impact of postnatal vitamin D supplementation in older infants whose 25(OH)D concentrations have reached a steady state.

Another strength of this analysis is that we were able to adjust for factors known to influence the composition of the microbiota of the infant, such as location and mode of delivery, type of infant feeding and number of older siblings and we excluded for two other determinants of intestinal microbiota, prematurity and exposure to antimicrobials [16].

A limitation of this study concerns the lower numbers of infants in the reference groups for *C*. *difficile* (n = 35) and *lactobacillus* spp. (n = 45) for the analyses involving vitamin D supplementation of the child or maternal vitamin D quintile. The number of infants not administered vitamin D supplementation was low because it is standard practice to supplement breast fed children with vitamin D, and the lower prevalence of colonization with these bacteria also contributed to this limitation.

Although we adjusted for the type of infant feeding in the maternal analyses, residual confounding may still be present because the infants with combined feeding were considered as an homogenous group. This group could theoretically represent infants ranging from those who were fed formula once a day to those who were breast fed only once a day.

Another limitation of our study is that no preservation or stabilization medium was added to the fecal samples during the transport to the laboratory. Theoretically this could have introduced bias in the bacterial composition due to the overgrowth of some bacteria or the DNA degradation of others. However, several studies have demonstrated a rather stable microbiota profile of fecal samples during storage at room temperature for up to 72 hours or more indicating that such bias might be limited [31–33].

Finally, the study is limited by the use of a single fecal sample which only represents a snapshot of the rapidly evolving microbiota composition. Inclusion of consecutive sampling would have been preferable.

To our knowledge, this is the first report of a large scale observational study indicating that prenatal vitamin D exposure, as measured either by maternal use of multivitamin supplements containing vitamin D, or 25(OH)D quintiles, influences several important bacterial taxa within the infant gut.

Confirmation of these findings is necessary because the identification of modifiable factors which shape the microbial population is important due to the immunomodulatory functions of the intestinal microbiota, which bestow it with the potential to significantly influence the development of disease or maintenance of health. The influence of postnatal vitamin D supplementation merits to be analyzed in a larger group of older infants and with extensive microbial profiling approach.

## Supporting information

S1 Fig Boxplots to describe log10 CFU of *Bifidobacterium* spp., *Escherichia coli*, *Bacteroides fragilis* group, *Clostridium difficile*, and *Lactobaccilus* spp. in infants colonized with the respective bacteria in relation to maternal multivitamin supplementation containing vitamin D. (DOCX)

S2 Fig Boxplots to describe log10 CFU of *Bifidobacterium* spp., *Escherichia coli*, *Bacteroides fragilis* group, *Clostridium difficile*, and *Lactobaccilus* spp. in infants colonized with the respective bacteria in relation to maternal 25(OH)D level (in quintiles). (DOCX)

S3 Fig Boxplots to describe log10 CFU of *Bifidobacterium* spp., *Escherichia coli*, *Bacteroides fragilis* group, *Clostridium difficile*, and *Lactobaccilus* spp. in infants colonized with the respective bacteria in relation to vitamin D supplement use of breastfed infants. (DOCX)
