# Supplementary material for: Influence of vitamin D on key bacterial taxa in infant microbiota in the KOALA Birth Cohort Study
Source: PLoS One. 2017 Nov 9;12(11):e0188011. doi: 10.1371/journal.pone.0188011 (PMC5679631; doi:10.1371/journal.pone.0188011)
Supplement: S1 Fig — (DOCX) [file pone.0188011.s001.docx]

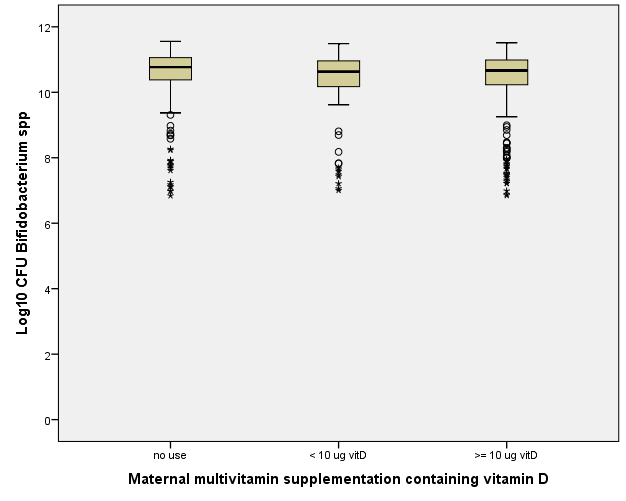

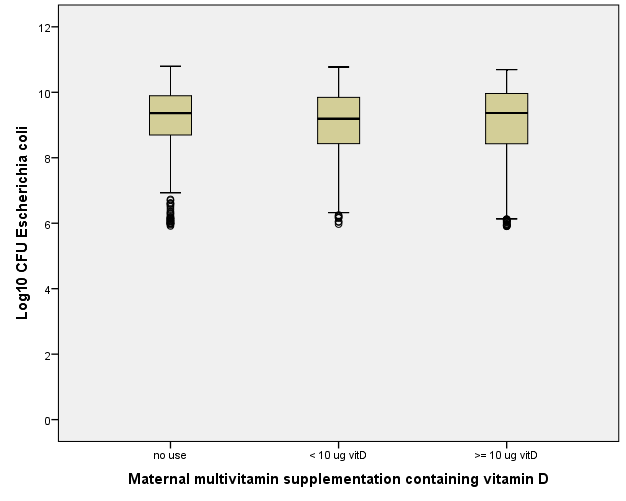


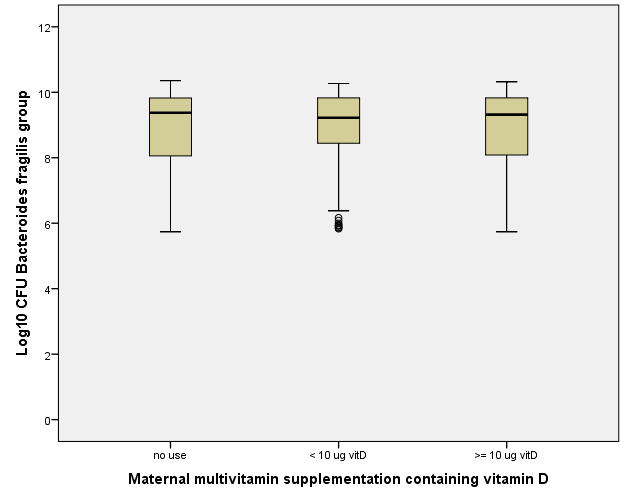

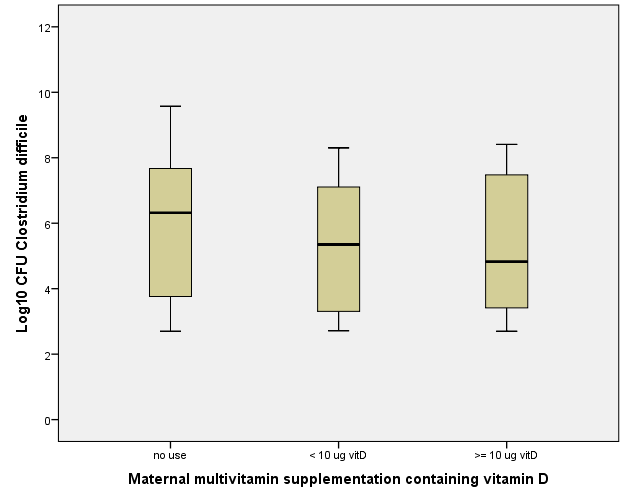


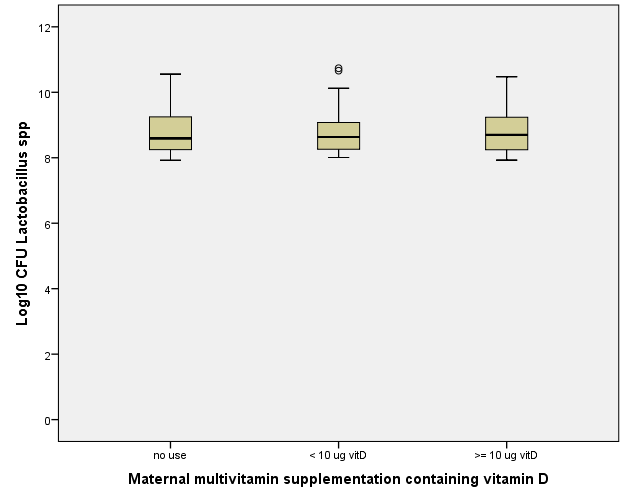


S1 Fig: Boxplots to describe log10 CFU of *Bifidobacterium* spp., *Escherichia coli*, *Bacteroides fragilis* group, *Clostridium difficile*, and *Lactobaccilus* spp. in infants colonized with the respective bacteria in relation to maternal multivitamin supplementation containing vitamin D
